# Associations between Serum Uric Acid and the Remission of Non-Alcoholic Fatty Liver Disease in Chinese Males

**DOI:** 10.1371/journal.pone.0166072

**Published:** 2016-11-11

**Authors:** Zhiwei Zhou, Kai Song, Jing Qiu, Yiying Wang, Chunxing Liu, Hui Zhou, Yunfang Xu, Zhirong Guo, Biao Zhang, Chen Dong

**Affiliations:** 1 Department of Epidemiology and Statistics, School of Public Health, Jiangsu Key Laboratory and Translational Medicine for Geriatric Disease, Medical College of Soochow University, Suzhou, Jiangsu, China; 2 Suzhou Industrial Park Centers for Disease Control and Prevention, Suzhou, China; 3 Huai’an Forth Hospital, Huai’an, China; East Tennessee State University, UNITED STATES

## Abstract

Epidemiological studies suggest that higher serum uric acid (sUA) level is significantly associated with nonalcoholic fatty liver disease (NAFLD) development. However, little information is available on the relationships between sUA and NAFLD remission. In the present study, 841 NAFLD males (30–75 years) were recruited from a Chinese prospective cohort study (PMMJS) and followed up for five years. The baseline sUA levels of participants were categorized into four quartiles: 191 μmol/L≤ sUA ≤ 347 μmol/L, 347 μmol/L < sUA ≤ 392 μmol/L, 392 μmol/L < sUA ≤ 441 μmol/L and 441 μmol/L<SUA≤676 μmol/L. As the results show, participants with elevated sUA levels at baseline were significantly associated with the decreased rate of NAFLD remission at the end of study (p<0.0001). After adjustment, RR (95%CI) for remitted NAFLD comparing Q1 to Q3 vs Q4 of sUA were 2.95 (1.49–5.83), 2.40 (1.22–4.73) and 1.39 (0.67–2.86), respectively. Furthermore, the sensitivity analysis showed these significant associations were not affected even after exclusion of participants who had hypertension, diabetes mellitus, MetS and hyperlipidemia. Additionally, the presence of the lowest quartile of sUA levels was still significantly associated with remitted NAFLD when the study population was stratified according to the smoking, and the median values of age, ALT, AST, serum creatinine, HDL-C and LDL-C. Therefore, our present study extended the previous findings and suggested that modulation of sUA levels may attenuate the progression of NAFLD.

## Introduction

Nonalcoholic fatty liver disease (NAFLD), characterized as abnormal lipid deposition in hepatocytes without excess alcohol intake, is a major cause of chronic liver disease worldwide. Approximately, 25.24% of the global population and 15–20% of Asian persons were affected by NAFLD. The disease refers to a pathological spectrum of physiological disorders ranging from lipid accumulation in hepatocytes (steatosis) to the development of superimposed inflammation (non-alcoholic steatohepatitis, NASH) and fibrosis leading to cirrhosis and hepatocellular carcinoma [1]. Over the last decade, there were growing evidences that the disease burdens of NAFLD were not only related to liver-related morbidity or mortality, but also related to the extrahepatic complications, including cardiovascular disease (CVD), chronic kidney disease (CKD) and cancer [2–4].

In physiological conditions, the level of serum uric acid (sUA) is maintained by the balance between uric acid production and excretion. Previously, more and more studies suggested that the sUA level was significantly elevated in chronic metabolic diseases, such as cardiovascular disease [5], type 2 diabetes mellitus (T2DM) [6] and metabolic syndrome (MetS) [7]. In 2002, Lonardo et al. firstly reported that the elevated sUA level was associated with an increased risk of NAFLD in Italian population [8]. After that, Liu et al. further observed that even in a normal range, each one mg increase of sUA could lead to 21% rise in NAFLD risk in Chinese population [9]. However, whether the level of sUA is associated with NAFLD remission remains unclear. Considering NAFLD is the leading cause of chronic liver disease in China and about 80% persons with NAFLD are males [10,11], we performed the present study to explore the potential links between the levels of sUA and the remissions of NAFLD in Chinese males.

## Materials and Methods

### Study population

The population included in the present study was recruited from the Wuxi center of the prospective cohort study of “The prevention of MS and multi-metabolic disorders in Jiangsu province of China” (PMMJS), which was established at 2003–2004 and aimed to investigate the epidemiologic characteristics of MetS and other chronic diseases, including hypertension and T2DM. The study design, protocol and sampling method have been described previously [12]. Briefly, total 5067 persons (aged 30–75 years) were recruited and completed the standard questionnaire at baseline (February 2003-August 2003). Between February and August 2007, 4661 (406 lost) individuals were accepted the first follow-up and received the hepatic ultrasound examination and 2009 persons were determined with fatty liver disease. In 2012, the participants with fatty liver disease were invited to participate in additional follow-up evaluation and 1947 persons (62 lost) completed. All of participants were asked to provide written informed consent and the study protocol was approved by the ethics committees of Soochow University.

**Fig 1:**
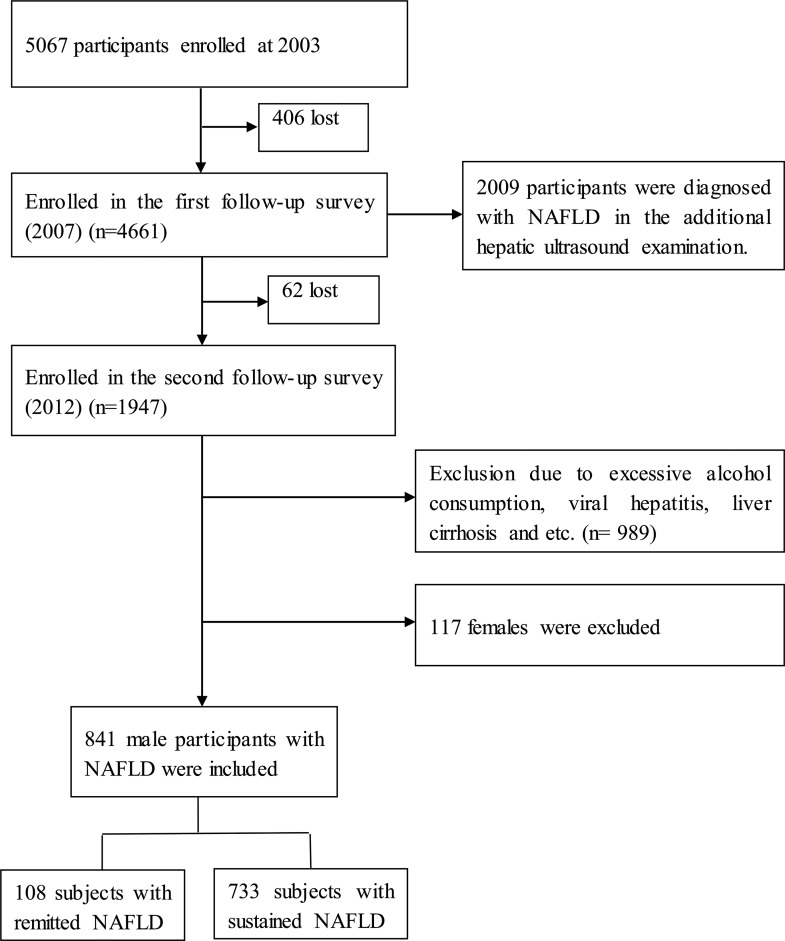
Study flow diagram for the included participants.

In the present analysis, the participants with fatty liver disease met the following criteria were excluded: (1) excessive alcohol consumption (≥ 30 g/day in men) (874); (2) liver cirrhosis or suspicion of malignancy (10), (3) HBsAg positive (104) or anti-HCV antibody positive (0), (4) schistosomiasis infection history (1); (5) 117 females. At last, a total of 841 males diagnosed with NAFLD were selected in the present study (Fig 1 and S1 Table).

### Data collection and biomedical measurement

In the present study, the information collected in 2007 included hepatic ultrasound examinations was used as baseline data. A face-to-face interview was carried out with all participants by a trained interviewer for the information about demographics (including age, gender, body weight, height and etc), smoking and drinking habits, main disease history and family history of hypertension, diabetes mellitus, obesity and hyperlipidemia and etc. Blood pressure was measured 3 times using a mercury sphygmomanometer in a seated position, with a 1 minute interval between measurements. Body mass index (BMI) was calculated by dividing weight in kilograms by height in meters squared.

After an overnight fast, a venous blood of 3 milliliter (ml) blood sample was obtained from each participant for biochemical analysis without freezing. The method for serum levels of uric acid detection was based on the Fossati enzymatic reaction using uricase with a Trinder-like end point. Additionally, the levels of fasting glucose (FPG), fasting serum triglycerides (TG), total cholesterol (TC), LDL-cholesterol (LDL-C), HDL-cholesterol (HDL-C), alanine aminotransferase (ALT), aspartate aminotransferase (AST) and γ-glutamyl transferase (γ-GT) were measured using commercial reagents on an autoanalyzer (Olypums AU640, Japan), respectively.

### Definitions

The diagnosis of NAFLD was based on the findings of hepatic ultrasound examination with a 3.5-MHz transducer (SoNoliNE Versa Plus, SIEMENS, Germany). According to the “Chinese Guideline on Diagnosis and Treatment of NAFLD” [13], the diagnosis of NAFLD should have the presence of at least 2 of the following 3 abnormal findings: diffuse increased echogenicity of liver relative to kidney, ultrasound beam attenuation, and poor visualization of intrahepatic structures. The ultrasonography was carried out by experienced radiologists who were blinded to the laboratory values of the examinees.

### Statistical analysis

Statistical analysis was performed with SAS 9.4 software (SAS Institute, Cary, NC, USA). The chi-square test was used to compare categorical variables between groups. For continuous variables, parameters that followed normal distribution were analyzed with *t*-test or ANOVA and expressed as the mean ± standard deviation (SD), median or percentage as suitable. In the present study, the participants were divided into four quartiles based on their SUA levels at baseline (191 μmol/L≤ sUA ≤ 347 μmol/L, 347 μmol/L < sUA ≤ 392 μmol/L, 392 μmol/L < sUA ≤ 441 μmol/L and 441 μmol/L<SUA≤676 μmol/L). The relative risk (RR) and 95% confidence intervals (CI) of NAFLD remission were calculated by quartiles of SUA. In the multivariate models, Model 1 was adjusted for age (years) and BMI (kg/m^2^), hypertension (yes), MetS (yes) and diabetes mellitus (yes). Model 2 was adjusted for model 1 plus ALT, AST and Scr. Model 3 was adjusted for model 2 plus TC, TG, HDL-C and LDL-C. All reported *P* values were two-tailed, and those <0.05 were considered statistically significant.

## Results

At the baseline, the mean age of studied NAFLD males was 53.51±7.71 years, and the mean sUA level was 395.25±72.85 μmol/L, respectively. The prevalence of current smoking, hypertension, MetS, and diabetes mellitus among the participants was 47.32%, 31.15%, 17.83% and 9.63%, respectively. Table 1 described the characteristics of studied participants according to their quartile measurements of sUA. Similar to the previous findings, participants with higher levels of sUA showed higher levels of serum ALT, AST, serum creatinine and TG, as well as BMI and WC when compared to those with lower sUA levels. However, the sUA levels were inversely associated with serum HDL-C levels and diabetes mellitus. No significant differences were found across sUA groups for the other variables such as age, current smoking, SBP, DBP and serum levels of FBG, TC, LDL-C and etc.

**Table 1:** Baseline characteristics of study subjects stratified by quartiles (Q) of sUA levels.

Variables	Quartiles of Serum Uric Acid				*P* value
	Q1(n = 211)	Q2(n = 210)	Q3(n = 210)	Q4(n = 210)	
Age (years)	54.0±7.2	54.0±7.5	53.1±7.8	53.0±7.9	0.3431
BMI (kg/m^2^)	26.0±2.4	26.0±2.5	26.1±2.4	26.7±2.4	0.0054
WC (cm)	89.8±6.1	89.9±6.3	89.9±6.4	91.4±6.3	0.0233
Current smokers (%)	109(51.7)	93(44.3)	99(47.1)	97 (46.2)	0.4791
Hypertension (%)	67(31.8)	62(29.5)	64(30.5)	69(32.9)	0.8908
Anti-Hypertension (%)	38(18.0)	36(17.1)	32(15.2)	33(15.7)	0.8620
Diabetes(%)	31(14.7)	18(8.6)	17(8.1)	15(7.1)	0.0360
Anti-Diabetes (%)	8(3.8)	9(4.3)	3(1.4)	8(3.8)	0.3554
SBP (mmHg)	119.6±11.9	119.4±11.6	120.8±12.0	121.7±12.0	0.1679
DBP (mmHg)	79.2±8.8	78.8±8.5	80.2±8.7	80.3±10.5	0.2168
FBG (mmol/L)	5.9±1.3	5.7±1.2	5.7±0.9	5.7±0.8	0.1380
ALT (U/L)	33.1±21.9	33.4±19.1	36.5±18.5	40.3±25.1	0.0014
AST (U/L)	24.6±9.4	24.6±7.4	26.2±8.1	27.8±10.0	0.0002
Serum creatinine (μmol/L)	74.3±11.1	76.9±10.1	78.7±9.6	81.7±10.4	< 0.0001
TC (mmol/L)	4.9±0.9	4.9±0.9	4.9±0.7	5.0±0.8	0.6974
TG (mmol/L)	1.9±1.5	2.2±2.0	2.3±1.3	2.6±1.6	0.0015
HDL-C (mmol/L)	1.2±0.2	1.1±0.2	1.1±0.3	1.1±0.2	< 0.0001
LDL-C (mmol/L)	3.1±0.8	3.1±0.8	3.1±0.7	3.1±0.8	0.9750
Anti-Hyperlipidemia (%)	32(15.2)	34(16.2)	36(17.1	35(16.7)	0.9541
Metabolic Syndrome (%)	36(17.06)	41(19.52)	34(16.19)	39(18.57)	0.8109

Numerical data were expressed as mean ± standard deviation or median. Categorical data were expressed as percentage.

In the present cohort study, the total rate of remitted NAFLD was 12.84% (108/841). In contrast to the participants with sustained NAFLD, the participants with remitted NAFLD were relatively lower BMI (P = 0.0039) and WC (P = 0.0006), as well as lower serum levels of AST (P = 0.0011), ALT (P <0.0001) and TG (P = 0.0293). In addition, among 150 participants with MetS, 19 (14.5%) persons have remitted from NAFLD at the end of study. As the results shown in Table 2, we did not observe the significant differences between the subjects with and without remitted NAFLD in age, current smoking, hypertension, diabetes mellitus, SBP, DBP, and the concentrations of FPG, serum creatinine, TC, HDL-C and LDL-C.

**Table 2:** Comparison of baseline parameters between subjects with remitted and sustained NAFLD.

	NAFLD		*P* value
	Remitted(n = 108)	Sustained(n = 733)	
Age (years)	54.7±7.7	53.3±7.7	0.0766
BMI (kg/m^2^)	25.6±2.3	26.3±2.5	0.0039
WC (cm)	88.3±5.9	90.5±6.3	0.0006
Current Smokers (%)	49(45.4)	349(47.6)	0.6631
Hypertension (%)	30(27.8)	232(31.7)	0.4172
Anti-Hypertension (%)	20(18.5)	119(16.2)	0.5508
Diabetes (%)	7(6.5)	74(10.1)	0.2346
Anti-Diabetes (%)	4(3.7)	24(3.3)	0.7739
SBP (mmHg)	118.9±10.5	120.6±12.1	0.1629
DBP (mmHg)	78.9±7.6	79.7±9.4	0.2764
FBG (mmol/L)	5.7±0.8	5.8±1.1	0.2752
ALT (U/L)	29.1±12.8	36.8±22.3	< .0001
AST (U/L)	23.8±6.1	26.1±9.2	0.0011
Serum creatinine (umol/L)	78.1±10.6	77.9±10.7	0.8829
TC (mmol/L)	4.9±0.8	4.9±0.9	0.8390
TG (mmol/L)	2.0±1.2	2.3±1.7	0.0293
HDL-C (mmol/L)	1.2±0.2	1.1±0.2	0.1287
LDL-C (mmol/L)	3.1±0.7	3.1±0.7	0.3344
Anti-Hyperlipidemia	17(15.7)	120(16.4)	0.8685
Metabolic Syndrome(%)	19(8.3)	131(17.9)	0.9436

Numerical data were expressed as mean ± standard deviation or median. Categorical data were expressed as percentage.

As the results shown, NAFLD were remitted in 18.96%, 16.19%, 9.52%, and 6.67% of studied males in the groups of 191 μmol/L≤ sUA ≤ 347 μmol/L, 347 μmol/L < sUA ≤ 392 μmol/L, 392 μmol/L < sUA ≤ 441 μmol/L and 441 μmol/L<SUA≤676 μmol/L, respectively. The increased levels of sUA at baseline were significantly associated with the decreased rate of NAFLD remission. This decreasing trend was especially prominent in the individuals with the highest sUA level (p < 0.0001).

As the results shown in Table 3, the RR (95% CI) for remitted NAFLD comparing Q1 to 3 vs Q4 were 3.28 (1.72–6.22), 2.70 (1.41–5.21), and 1.47 (0.72–3.00) in unadjusted logistic models, respectively. After adjusting for age, BMI, hypertension, MetS and diabetes mellitus, the RR (95% CI) for NAFLD remission in the Q1 compared with Q4 was 3.12 (1.63–5.99). The significant association was not affected after adjusting for the additional variables including ALT, AST, serum creatinine, TG, TC, HDL-C, LDL-C and anti-hypertension, anti-diabetes and anti-hyperlipidemia medicine (RR _Q1 vs Q4_, 3.16; 95% CI, 1.59–6.28).

**Table 3:** RR (95% CI) for remission of patients with NAFLD according to SUA quartiles.

	Quartiles of Serum Uric Acid			
	Q1(n = 211)	Q2(n = 210)	Q3(n = 210)	Q4(n = 210)
Remission cases	40	34	20	14
Remission rate (%)	18.96	16.19	9.52	6.67
Unadjusted	3.28 (1.72, 6.22)	2.70 (1.41, 5.21)	1.47 (0.72, 3.00)	1.00 (Ref)
Model 1	3.12 (1.63, 5.99)	2.46 (1.27, 4.77)	1.40 (0.68, 2.86)	1.00 (Ref)
Model 2	3.20 (1.62, 6.29)	2.47 (1.25, 4.86)	1.44 (0.70, 2.97)	1.00 (Ref)
Model 3	3.06 (1.54, 6.08)	2.38 (1.21, 4.71)	1.40 (0.68, 2.90)	1.00 (Ref)
Model 4	3.16 (1.59, 6.28)	2.40 (1.22, 4.75)	1.44 (0.69, 2.97)	1.00 (Ref)

Data were expressed as odds ratios (95%CI) by univariate and multivariate logistic regression analysis. Regression models were adjusted as follows: Model 1: adjusted for age, BMI, hypertension, diabetes and MetS; Model 2: Model 1 plus ALT, AST and serum creatinine; Model 3: Model 2 plus TC, TG, HDL-C and LDL-C; Model 4: Model 3 plus Anti-hypertension, Anti-diabetes and Anti- hyperlipidemia.

Q1: 191 μmol/L≤ sUA ≤347 μmol/L; Q2: 347 μmol/L< sUA ≤392 μmol/L

Q3: 392 μmol/L< sUA ≤441 μmol/L; Q4: 441 μmol/L< sUA ≤676 μmol/L.

Table 4 showed subgroup analysis for remitted NAFLD of Q1, Q2 and Q3 using the Q4 as the reference. Compared to the prevalence of NAFLD remission in the highest quartile of sUA (Q4), the presence of the lowest quartile of sUA levels (Q1) was significantly associated with remitted NAFLD even when the study population was stratified according to the smoking, and the median values of age, ALT, AST, serum creatinine, HDL-C and LDL-C. Moreover, subjects had a significantly higher RR _Q1 vs Q4_ in subgroups wherein BMI > 26 kg/m2, TC ≤ 4.84 mmol/L, TG ≤ 1.87 mmol/L, non-hypertension and non-MetS.

**Table 4:** Stratified analysis of sUA and NAFLD remission in different subgroups (n = 841).

Subgroups	N(%)	Q1(n = 211)	Q2(n = 210)	Q3(n = 210)	Q4(n = 210)
Age(years)					
≤56.0	49 (10.36)	2.66 (1.05, 6.72)	2.20 (0.86, 5.63)	1.05 (0.37, 2.96)	1.00 (Ref)
>56.0	59 (16.03)	3.14 (1.10, 8.97)	2.58 (0.92, 7.22)	1.75 (0.60, 5.10)	1.00 (Ref)
BMI(kg/m^2^)					
≤26.0	63 (15.25)	1.69 (0.69, 4.15)	1.36 (0.54, 3.40)	0.96 (0.37, 2.46)	1.00 (Ref)
>26.0	45 (10.51)	5.80 (1.91, 17.61)	4.87 (1.65, 14.40)	2.29 (0.71, 7.44)	1.00 (Ref)
Current smokers					
No	59 (13.32)	3.79 (1.34, 10.71)	4.91 (1.82, 13.29)	1.35 (0.44, 4.13)	1.00 (Ref)
Yes	49 (12.31)	2.37 (0.93, 6.06)	0.83 (0.29, 2.45)	1.40 (0.52, 3.71)	1.00 (Ref)
Hypertension					
No	78 (13.47)	3.30 (1.44, 7.57)	2.01 (0.86, 4.67)	1.55 (0.64, 3.76)	1.00 (Ref)
Yes	30 (11.45)	2.00 (0.54, 7.45)	4.00 (1.17, 13.67)	1.10 (0.28, 4.26)	1.00 (Ref)
Anti-hypertension					
No	88 (12.54)	3.13 (1.45, 6.71)	2.06 (0.95, 4.45)	1.35 (0.60, 3.07)	1.00 (Ref)
Yes	20 (14.39)	1.46 (0.26, 8.18)	3.63 (0.70, 18.97)	1.68 (0.29, 9.70)	1.00 (Ref)
ALT(U/L)					
≤30.0	69 (15.79)	2.94 (1.22, 7.08)	2.07 (0.86, 4.97)	1.26 (0.49, 3.22)	1.00 (Ref)
>30.0	39 (9.65)	3.29 (1.03, 10.45)	3.18 (1.02, 9.87)	1.66 (0.50, 5.49)	1.00 (Ref)
AST(U/L)					
≤23.0	59 (13.79)	2.83 (1.08, 7.38)	2.20 (0.86, 5.65)	1.04 (0.35, 3.05)	1.00 (Ref)
>23.0	49 (11.86)	2.68 (0.98, 7.39)	2.62 (0.95, 7.23)	1.63 (0.60, 4.45)	1.00 (Ref)
Serum creatinine (μmol/L)					
≤77.7	54 (12.77)	3.42 (1.11, 10.67)	2.14 (0.66, 6.94)	1.59 (0.46, 5.47)	1.00 (Ref)
>77.7	54 (12.92)	2.79 (1.08, 7.20)	2.67 (1.11, 6.42)	1.29 (0.51, 3.29)	1.00 (Ref)
TC(mmol/L)					
≤4.8	53 (12.56)	3. 38 (1.20, 9.56)	2.73 (0.96, 7.77)	1.52 (0.51, 4.57)	1.00 (Ref)
>4.8	55 (13.13)	2.50 (0.99, 6.33)	2.12 (0.83, 5.41)	1.21 (0.45, 3.26)	1.00 (Ref)
TG(mmol/L)					
≤1.9	59 (13.88)	3.73 (1.30, 10.70)	3.07 (1.06, 8.86)	0.94 (0.27, 3.24)	1.00 (Ref)
>1.9	49 (11.78)	2.54 (0.97, 6.63)	2.01 (0.78, 5.14)	1.91 (0.76, 4.78)	1.00 (Ref)
HDL-C(mmol/L)					
≤1.1	52 (12.29)	2.50 (1.04, 6.03)	2.54 (1.09, 5.90)	0.86 (0.32, 2.30)	1.00 (Ref)
>1.1	56 (13.40)	3.60 (1.09, 11.93)	2.20 (0.64, 7.55)	2.50 (0.74, 8.45)	1.00 (Ref)
LDL-C(mmol/L)					
≤3.0	50 (11.79)	2.71 (0.91, 8.08)	2.52 (0.86, 7.39)	1.43 (0.44, 4.62)	1.00 (Ref)
>3.0	58 (13.91)	2.79 (1.10, 7.03)	2.14 (0.85, 5.41)	1.16 (0.44, 3.05)	1.00 (Ref)
Anti-Hyperlipidemia					
No	91 (12.93)	3.96 (1.77, 8.83)	3.39 (1.52, 7.56)	1.78 (0.75, 4.19)	1.00 (Ref)
Yes	17 (12.41)	0.57 (0.09, 3.57)	0.36 (0.06, 2.41)	0.37 (0.06, 2.52)	1.00 (Ref)
Metabolic Syndrome					
No	89 (12.88)	3.13 (1.47, 6.68)	2.08 (0.96, 4.50)	1.21 (0.53, 2.75)	1.00 (Ref)
Yes	19 (12.67)	1.63 (0.25, 10.78)	4.20 (0.84, 21.15)	3.09 (0.59, 16.25)	1.00 (Ref)

Data were expressed as RR (95%CI). Analysis according to the medium value of Age, BMI, ALT, AST, serum creatinine, TC, TG, HDL-C and LDL-C, and current smokers (yes/no), hypertension (yes/no), Anti-hypertension (yes/no), Anti-hyperlipidemia(yes/no) and MetS (yes/no). When compared in different groups, the RR was adjusted for other covariates.

In order to avoid the effects of anti-hypertension, anti-diabetes and anti-hyperlipidemia medicine on the NAFLD remission in the present study, we performed the sensitivity analysis to further evaluate the potential links between the sUA levels and NAFLD remission. As expected, we observed the similar significant associations between sUA and NAFLD remission as the results showed above (RR _Q1 vs Q4_, 3.07; 95% CI, 1.26–7.48) (Table 5). Moreover, further analysis suggested that exclusion of NAFLD males who had hypertension, diabetes mellitus, MetS and hyperlipidemia did not alter the association between sUA and NAFLD remission (RR _Q1 vs Q4_, 3.36; 95% CI, 1.03–10.99) (S2 Table).

**Table 5:** Sensitivity analysis on the associations between sUA levels and NAFLD remission (n = 516)*.

	Quartiles of Serum Uric Acid			
	Q1(n = 129)	Q2(n = 129)	Q3(n = 129)	Q4(n = 129)
Remission cases	29	18	12	8
Remission rate (%)	22.48	13.95	9.30	6.20
Unadjusted	4.39 (1.92, 10.02)	2.45 (1.03, 5.86)	1.55 (0.61, 3.93)	1.00 (Ref)
Model 1	3.86 (1.67, 8.93)	2.06 (0.85, 5.01)	1.46 (0.57, 3.73)	1.00 (Ref)
Model 2	3.37 (1.40, 7.92)	1.80 (0.73, 4.45)	1.40 (0.54, 3.63)	1.00 (Ref)
Model 3	3.07 (1.26, 7.48)	1.72 (0.69, 4.31)	1.35 (0.52, 3.51)	1.00 (Ref)

*Sensitivity analysis on the associations between sUA levels and NAFLD remission after excluded the participants who had anti-hypertension, anti-diabetes and anti-hyperlipidemia medicine.

Data were expressed as odds ratios (95%CI) by univariate and multivariate logistic regression analysis. Regression models were adjusted as follows: Model 1: adjusted for age, BMI, hypertension, diabetes and MetS; Model 2: Model 1 plus ALT, AST and serum creatinine; Model 3: Model 2 plus TC, TG, HDL-C, LDL-C.

Q1: 191 μmol/L≤ sUA ≤347 μmol/L; Q2: 347 μmol/L< sUA ≤392 μmol/L

Q3: 392 μmol/L< sUA ≤441 μmol/L; Q4: 441 μmol/L< sUA ≤676 μmol/L.

## Discussion

Currently, NAFLD represents an important burden of chronic liver disease worldwide [14,15]. Similar to other countries, the prevalence of NAFLD is much higher in males in China, being 13.3% and 2.7% in males and females, respectively [11]. In addition, it is well known that hyperuricemia affects males more commonly than females [16,17], it is therefore clear that the need for the investigation on the relationships between sUA levels and NAFLD remission in males, is critical.

To our knowledge, the present study is the largest prospective cohort study performed to evaluate the potential links between sUA levels and NAFLD remission in Chinese males. As expected, our results showed that the sUA concentrations were inversely associated with NAFLD remission. The group with higher sUA concentration at baseline showed lower remission rates of NAFLD. Previously, the association between sUA and NAFLD development has been widely discussed. For instance, a Korean epidemiological study reported that the OR of NAFLD in the highest quartile were 1.51 (1.31–1.73) in males compared with the lowest sUA quartile [18]. A cross-sectional study conducted in the United States involving 5370 NHANS (1988–1994) participants also suggested that higher sUA levels were significantly associated with NAFLD development, and the multivariate OR comparing Q5 of sUA (7.1–11.4 mg/dL) with Q1 (1.7–5.1 mg/dL) was 1.7 (1.1–2.5) [19]. A population-based study, which observed 60,455 subjects in China, reported that the adjusted ORs comparing the highest quartile of sUA (> 311 μmol/L) with the lowest quartile (≤ 230 μmol/L) was 1.887 (1.718–2.072) [20]. In addition to these studies, several meta-analysises further indicated that increased sUA levels are independently associated with NAFLD occurrence regardless of gender, age, obesity, and MetS [21,22]. Therefore, our present study extends the previous findings, suggesting that intervention to lower SUA levels may reduce the risk of development and progression of NAFLD.

A variety of mechanisms could explain the inverse associations of sUA levels with NAFLD remission, including insulin resistance, hyperuricemia-induced endothelial dysfunction, oxidative and inflammatory damages [23–25]. In an animal experiment, Garcia-Ruiz reported that treatment with uric acid in obese ob/ob mice could lead to a nearly complete resolution of fatty liver, indicating that downregulation of uric acid might play a protective role in NAFLD [25]. In addition, Lanaspa et al. found that sUA could directly stimulate hepatic fat accumulation, and identified a mechanism that might involve the translocation of NADPH oxidase to the mitochondria with subsequent inactivation of aconitase, accumulation of citrate, and stimulation of fat synthesis [23].

Although the results were controversy in the previous literature, accumulating evidences indicated that elevated level of sUA was significantly associated with the development and the adverse outcomes of hypertension and MetS [26–28]. Therefore, it was not surprise results that the remission rates of NAFLD were not different between the groups of lowest and highest quartile of sUA among the NAFLD males with hypertension, or those with higher level of TC and TG. Additionally, we observed that among the NAFLD males with lower BMI, the rate of NAFLD remission in the lowest quartile of sUA was not different with those in the highest quartile of sUA. Considering uric acid could act as either antioxidant or prooxidant depending on its circumstances, especially on the availability of lipid hydroperoxides [29], the mechanism of the effects of sUA on NAFLD remission in non-obese population should be further investigated.

Similar to the previous studies, our present study was not without limitations. First, the diagnosis of NAFLD was made by ultrasonography examination but not by liver biopsy, although studies using ultrasound and biopsy supported that ultrasonographic results reliably predict fatty liver [30,31]. Second, we only analyzed the association between sUA and NAFLD remission in Chinese males in the present study. More studies should be performed to evaluate the effects of sUA on the remitted NAFLD in females or other racial population. Last, the previous studies have already indicated that the NAFLD is significantly associated with hypertension, diabetes mellitus and MetS [5–7], whether the remission of NAFLD is benefit for these chronic diseases should be discussed in future.

## Conclusion

In summary, our present results provided the new evidences that the concentrations of sUA were inversely associated with NAFLD remission in Chinese males. It suggests that modulation of sUA levels may benefit for the NAFLD remission and attenuate the disease severity and progression.

## Supporting Information

S1 Table Origiginal information of the participants included in the present study. (XLSX)

S2 Table Sensitivity analysis on the associations between sUA levels and NAFLD remission (n = 282)*. (DOCX)
