# Supplementary material for: Associations between Serum Uric Acid and the Remission of Non-Alcoholic Fatty Liver Disease in Chinese Males
Source: PLoS One. 2016 Nov 11;11(11):e0166072. doi: 10.1371/journal.pone.0166072 (PMC5106003; doi:10.1371/journal.pone.0166072)
Supplement: S2 Table — (DOCX) [file pone.0166072.s002.docx]

S2 Table Sensitivity analysis on the associations between sUA levels and NAFLD remission (n=282)

|  | Quartiles of Serum Uric Acid | | | |
| --- | --- | --- | --- | --- |
|  | Q1(n=71) | Q2(n=70) | Q3(n=71) | Q4(n=70) |
| Remission cases | 17 | 13 | 6 | 5 |
| Remission rate (%) | 23.94 | 18.57 | 8.45 | 7.14 |
| Unadjusted | 4.09 (1.42, 11.82) | 2.97 (0.99, 8.83) | 1.20 (0.35, 4.13) | 1.00 (Ref) |
| Model 1 | 4.11 (1.38, 12.28) | 2.95 (0.94, 9.22) | 1.21 (0.35, 4.26) | 1.00 (Ref) |
| Model 2 | 3.49 (1.13, 10.81) | 2.88 (0.89, 9.32) | 1.25 (0.34, 4.50) | 1.00 (Ref) |
| Model 3 | 3.36 (1.03, 10.99) | 2.84 (0.84, 9.55) | 1.20 (0.32, 4.46) | 1.00 (Ref) |

Data were expressed as odds ratios (95%CI) by univariate and multivariate logistic regression analysis. Regression models were adjusted as follows: Model 1: adjusted for Age, BMI, SBP, DBP and Glucose; Model 2: Model 1 plus ALT, AST and Serum creatinine; Model 3: Model 2 plus TC, TG, HDL-C, LDL-C.

Q1: 191 μmol/L≤ sUA ≤347 μmol/L; Q2: 347 μmol/L< sUA ≤392 μmol/L;

Q3: 392 μmol/L< sUA ≤441 μmol/L; Q4: 441 μmol/L< sUA ≤676 μmol/L.
